# P39 Bloodstream infections (BSI) in tertiary ICU—an update!

**DOI:** 10.1093/jacamr/dlac004.038

**Published:** 2022-02-16

**Authors:** Veer M. Patel, Seema Desai

**Affiliations:** Keele University School of Medicine, Keele ST5 5BG, UK; University Hospitals of North Midlands, Stoke-on-Trent ST4 6QG, UK

## Abstract

**Background:**

Patients in ICU represent a small proportion of inpatients; however they account for up to 25% of all hospital acquired infections (HAI) and are 2–5 times more likely to develop a HAI than the average patient. Management of bloodstream infections (BSI) in ICU is a rising problem due to increasing antibiotic resistance posing challenges to antimicrobial stewardship (AMS) leading to higher mortality in ITU patients. It increases duration of inpatient stay, hospital costs and worsens prognosis.

**Objectives:**

To find all-cause mortality in patients with BSI admitted to intensive care at 14 and 28 days from admission.

**Methods:**

We used retrospective patient data from the hospital database between March 2019 to March 2020.

**Results:**

We included 113 patients admitted to ICU and had BSI, of which 12.4% died at 14 days and 24.8% died at 28 days with overall mortality of 33.6% at discharge. Respiratory comorbidity was found significantly higher (48.1%). Patients with Gram-negative BSI had higher mortality with 14.29% at 14 days when compared with Gram-positive BSI, but 28 day mortality was similar in both groups (25.71% and 25% respectively), which is concerning.

**Conclusions:**

This study concludes that patients admitted to ICU have higher mortality at 28 days irrespective of the pathogen isolated in the bloodstream. This could be explained due to prolonged stay in ICU leading to increased burden from HAI. Future AMS will have to focus on a prevention model for this cohort to improve patient outcome.

